# Supplementary material for: The Extraction Using Deep Eutectic Solvents and Evaluation of Tea Saponin
Source: Biology (Basel). 2024 Jun 14;13(6):438. doi: 10.3390/biology13060438 (PMC11201205; doi:10.3390/biology13060438)
Supplement: Supplementary file 1 [file biology-13-00438-s001.zip › biology-3020740-supplementary.pdf]

Supplementary

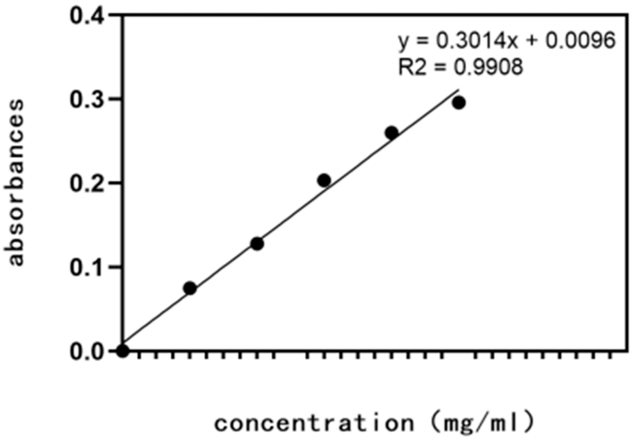

Figure S1. Tea Saponin Standard Curve Chart in vanillin-sulfuric acid method.

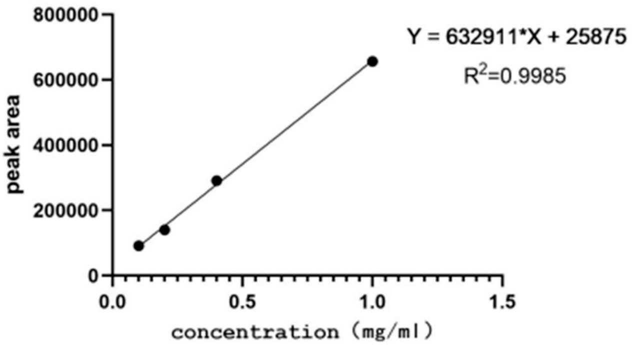

Figure S2. Tea Saponin Standard Curve Chart in HPLC.

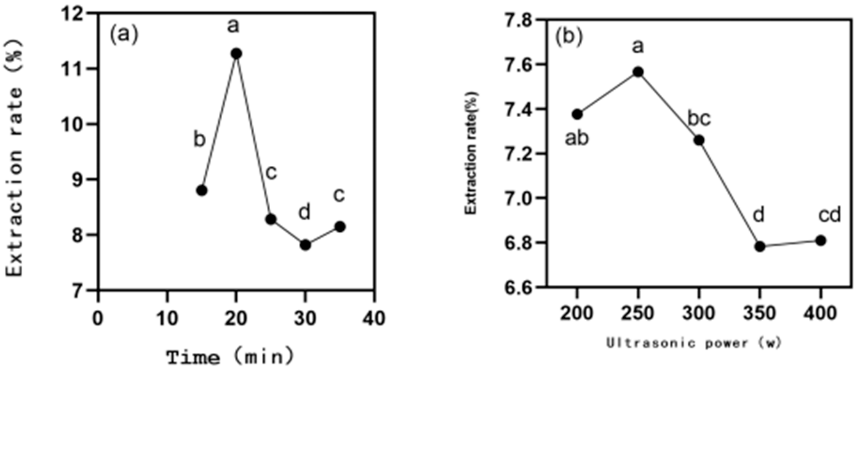

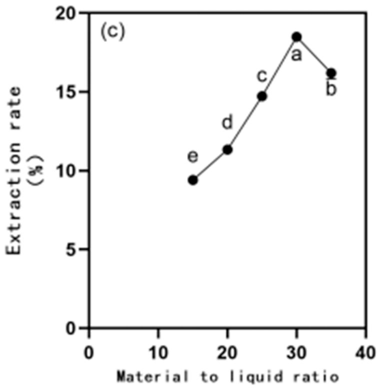

Figure S3. Single factor experimental result chart. (a) a single-factor experiment for ultrasound time. (b) a single-factor experiment for ultrasound power. (c) a single-factor experiment for material-to-liquid ratio.

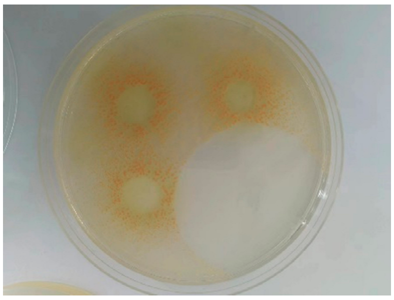

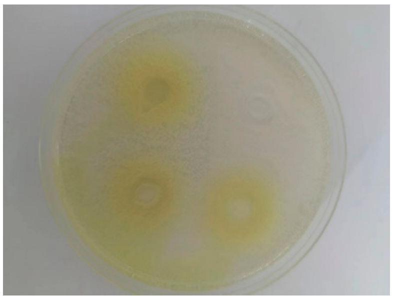

E. coli *S. pneumoniae R*

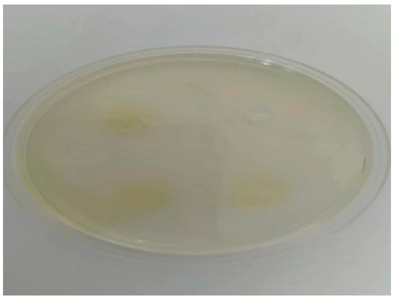

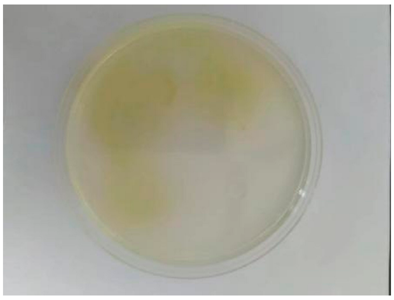

Enterococcus faecalis *Staphylococcus aureus*

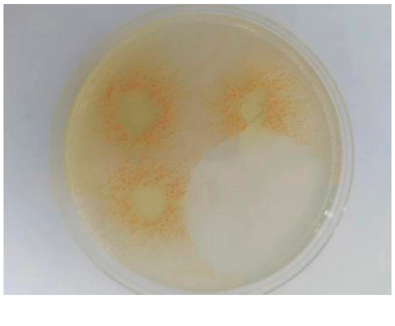

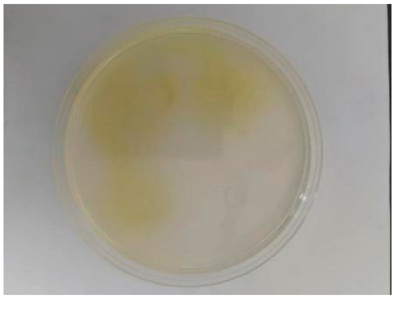

Bacillus subtilis *Pseudomonas aeruginosa*

Figure S4. Circle of inhibition test

Ampicillin was used as a control, and tea saponin was diluted to 1.5 mg/mL, 0.75 mg/mL and 0.375 mg/mL, respectively, and the diameters of the inhibition circles were measured after incubation for a period of time

Table S1. Orthogonal Tables.

	A extraction time (min)	B material-to-liquid ratio	C ultrasonic power (w)	D	rate
1	1	1	1	1	13.781
2	1	2	2	2	13.077
3	1	3	3	3	12.147
4	2	1	2	3	9.379
5	2	2	3	1	16.348
6	2	3	1	2	18.090
7	3	1	3	2	17.983
8	3	2	1	3	20.926
9	3	3	2	1	10.311
K1	39.004	41.142	52.797	40.439	
K2	43.816	50.351	32.766	49.149	
K3	49.220	40.547	46.477	42.452	
k1	13.001	13.714	17.599	13.480	
k2	14.605	16.784	10.922	16.383	
k3	16.407	13.516	15.492	14.151	
R	10.215	9.804	20.030	8.710	

Table S2. Analysis of variance results.

Source of variance	sum of squared deviations	degrees of freedom	mean square	F-value	P-value	significance
A	0.001741222	2	0.000870611	5.651688854	0.15033776	Not significance
B	0.002014305	2	0.001007153	6.538066067	0.132660021	Not significance
C	0.006990446	2	0.003495223	22.68970497	0.042212429	significance
D	0.0013864	9	0.000154044	1	0.5	
